# Potentiometric Sensor Based on Carbon Paste Electrode for Monitoring Total Residual Chlorine in Electrolytically-Treated Ballast Water

**DOI:** 10.3390/s21020350

**Published:** 2021-01-07

**Authors:** Yaning Zhang, Zhihui Li, Xiaotong Guo, Guangzhou Liu, Shuyong Zhang

**Affiliations:** 1 School of Chemistry and Chemical Engineering, Shandong University, Jinan 250100, China; yaningzhang@mail.sdu.edu.cn; 2 Institute of Marine Science and Technology, Shandong University, Qingdao 266237, China; 201712011@mail.sdu.edu.cn (Z.L.); 201611543@mail.sdu.edu.cn (X.G.); liuguangzhou@sdu.edu.cn (G.L.)

**Keywords:** ballast water, electrolytic treatment, carbon paste electrode, total residual chlorine, potentiometric sensor

## Abstract

A new potentiometric sensor based on modified carbon paste electrode (CPE) was prepared for the sensitive and selective detection of total residual chlorine (TRC) in simulated electrolytically-treated ballast water (BW). The modified CPE was prepared using ferrocene (Fc) as the sensing species and paraffin oil as the binder. It is revealed that the addition of Fc can significantly shorten the response time and improve the reproducibility, selectivity, and stability of the sensor. The open circuit potential of the Fc-CPE is in linear proportion to the logarithm of TRC within the TRC concentration range from 1 mg∙dm^−3^ to 15 mg∙dm^−3^. In addition, the Fc-CPE sensor exhibits good selectivity to TRC over a wide concentration range of the possible co-exiting interference ions in seawater. The Fc-CPE electrode can be used as a convenient and reliable sensor for the continuous monitoring of TRC during the electrolytic treatment of BW.

## 1. Introduction

At present, ocean transportation plays a key role in world trade. In the process of ocean transportation, ballast water (BW) is usually used to ensure the stability of the ships. Every year, about 10 billion tons of BW with many kinds of aquatic lives are transferred to different places all around the world [1,2,3,4]. The random discharge of BW with bacteria, viruses, and pathogens may cause ecological disasters and threaten human health, which has attracted great attention from the International Maritime Organization (IMO) and governments in recent years [5,6]. A number of technologies and rules for BW treatment have been established so as to eliminate the possible invasion of alien marine species. Among these methods, electrolytical treatment [5,7,8] is adopted as the most effective, convenient, and promising method due to its advantages, such as low price, high efficiency, no addition of chemical reagents, and safety, as well as the long-term stability. During electrolytical treatment, chlorine generated on the anode (Equation (1)) reacts with sodium hydroxide generated on the cathode, forming sodium hypochlorite (Equation (2)). Then, hypochlorous acid may form due to hydrolysis (Equation (3)).

(1) $${2\mathrm{NaCl}\;+\;2H}_{2}O\overset{\mathrm{electrolysis}}{\to }2\mathrm{NaOH}+{\mathrm{Cl}}_{2}+H_{2}$$

(2) $$2\mathrm{NaOH}\;+{\mathrm{Cl}}_{2}\overset{}{\to }{\mathrm{NaCl}\;+\;\mathrm{NaClO}\;+\;H}_{2}O$$

(3) $${\mathrm{NaClO}\;+\;H}_{2}O\overset{}{\to }\mathrm{HClO}\;+\;\mathrm{NaOH}$$

Therefore, there are Cl_2_, NaClO, and HClO present in the electrolytically-treated BW, which are all effective for killing marine species. The total concentration of these active species is termed as the total residual chlorine (TRC).

The sterilization capacity of the electrolytic treatment depends on the TRC concentration. It is found that the sterilization is not sufficient when TRC concentration is less than 5 mg∙dm^−3^. On the contrary, when the TRC concentration in the electrolytically-treated BW is higher than 15 mg∙dm^−3^, the strong oxidation of the solution may do harm to the environment of the discharged area and cause serious corrosion of the ship steel. The optimal concentration of TRC is found ranging between 6–10 mg∙dm^−3^ [8,9]. Therefore, to develop a continuous online method to monitor the TRC concentration in BW during electrolysis, to automatically switch on or turn off the electrolyzer when the TRC is less than 5 mg∙dm^−3^ or reaches 10 mg∙dm^−3^, so as to keep the TRC concentration within the optimal range, is necessary for application of the electrolytic treatment method.

Up to now, various analytical methods for determination of TRC, such as spectrophotometric methods [10], titration [11], chromatography coupled to mass spectrometry [12], chemiluminescence [13,14], and electrochemical methods [15,16,17,18], have been established. The spectrophotometric methods based on o-toluidine (DMB) [19] and N, N-diethyl-p-phenylenediamine (DPD) [20,21] and titration method are widely used for detection of TRC in water. However, the complicated sampling and treating procedures of these spectrophotometric methods, titration, and chemiluminescence methods make them unsuitable for the continuous online monitoring of TRC. Electrochemical methods include the galvanometric methods and the potentiometric methods. Numerous galvanometric sensors using different electrode materials, including screen-printed electrochemical sensor [22], platinum, gold, glassy carbon [23], graphite [24], Prussian blue [15], poly MnTAPP-nano Au [25], carbon [26], polydopamine [27], and multi-walled carbon nanotubes [28], for monitoring TRC have been reported. Although these galvanometric methods perform quite well for TRC measurement in tap water, it is not suitable for detection of TRC in the electrolytically-treated BW due to the large deviation and unstable current arising from the differences in temperature, composition, and conductivity of the BW at different locations. Compared with galvanometric sensors, potentiometric sensors are more attractive for detecting TRC concentration due to the advantages of simple operation, low cost, and continuous online detection. A potentiometric sensor using ferrocene-enhanced polyvinyl chloride-coated glassy carbon electrode [29] was fabricated for continuous monitoring of TRC without sampling and treating procedures. However, the slow response speed, poor potential stability, and easy stripping of polymer film make the ferrocene-enhanced polyvinyl chloride-coated glassy carbon electrode deteriorate within 7 days. Although some other simple all-solid-state potentiometric sensors using Pt, Al, or stainless steel [30] for the online monitoring of residual chlorine in tap water have been reported, the sensitivity in higher pH region and long-term stability of them are not satisfactory.

In 1958, Adams [31] reported a novel kind of electrode composed of carbon powder and non-electroactive materials, which is known as the first report of carbon paste electrode (CPE). CPE with large specific area and optimal pore structure is a convenient conductive substrate for preparation of chemically-modified electrode. It has attracted much attention due to its simple structure, low cost, easy regeneration, rapid electrochemical response, and low ohmic resistance [32]. In recent years, a lot of electrochemical sensors based on chemically-modified carbon paste electrode have been established for analysis of organic substances and drugs such as ascorbic acid, antipsychotic, and olanzapine [33]. It has been also used for detection of a number of inorganic cations, such as Ag^+^ [34], Hg^2+^ [35], Cd^2+^ [36], and Cu^2+^ [37]. Only a few researches on detection of anions haven been reported. To our knowledge, there is no report on detection of TRC based on CPE.

In this work, a new potentiometric sensor based on carbon paste electrode modified by ferrocene (Fc) is prepared for detecting TRC in simulated electrolytically-treated BW. The performance of the Fc-CPE sensor is studied.

## 2. Materials and Methods

### 2.1. Reagents and Materials

Graphite powder (GP) (Aladdin Industrial Co. Ltd., Shanghai, China) and paraffin oil (PO) (Fuyu Fine Chemical Co. Ltd., Tianjin, China) of high purity were used for the preparation of the carbon pastes. Ferrocene, tetrahydrofuran (THF), Na_2_SO_4_, MgCl_2_, NaHCO_3_, NaBr, KCl, and CaCl_2_ were all analytical reagents. The concentration of the commercial sodium hypochlorite solution (Dekang Chemical Co. Ltd., Laiyang, China) with TRC ≥ 10% was calibrated using iodometric titration. Then, a stock solution containing 50 mg∙dm^−3^ TRC was prepared by diluting the commercial sodium hypochlorite solution with 3.5 wt.% NaCl solution. After calibration of the stock solution, the simulated electrolytically-treated BW solutions with TRC of 0 mg∙dm^−3^, 1 mg∙dm^−3^, 2 mg∙dm^−3^, 5 mg∙dm^−3^, 10 mg∙dm^−3^, and 15 mg∙dm^−3^ were prepared by diluting the 50 mg∙dm^−3^ stock solution with 3.5 wt.% NaCl solution. All the solutions were stored in brown bottle, placed in a dark place, and calibrated regularly. Ultrapure water (Millipore, 18.25 M Ω cm) was used for preparation of all solutions.

### 2.2. Preparation of the Fc-Modified Carbon Paste Electrode (Fc-CPE)

**Figure 1:**
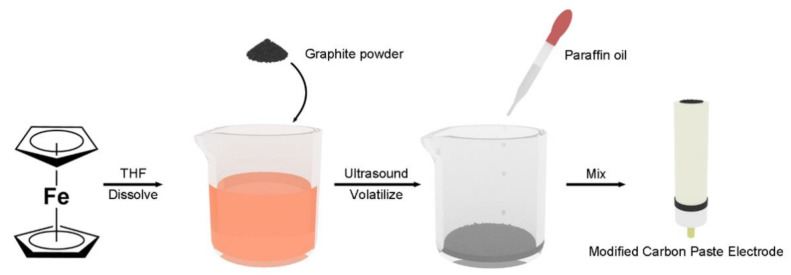
The preparation process of modified carbon paste electrode.

For preparation of the Fc-modified carbon paste electrode, different amounts of active material (Fc) were dissolved in 2 mL THF. Then, different amounts of graphite powder were added into the solution with the aid of ultrasonic for 2 min. After complete volatilization of the solvent, the Fc-modified carbon powder was obtained. The Fc-modified carbon powder was then mixed with different amounts of Paraffin oil, using a mortar, until a homogeneous carbon paste was obtained. The composition of the carbon paste is summarized in Table 1. A portion of the carbon paste mixture was packed carefully into the glass tube (3 mm in diameter) (Figure 1). The electrical contact to the carbon paste was made with a copper wire. After polishing on a weighing paper, a smooth, fresh surface of the carbon paste electrode was achieved. The newly-made carbon paste electrode was stored for ca. 24 h before use. The storage can help the electrode to achieve final homogenization, ensuring better reproducibility and stability [38]. The CPE without Fc modification was also made using neat graphite powder, following the same procedure, for control experiment.

Prior to each measurement, the electrode was activated by immersing in 3.5 wt.% NaCl solution for 40 min and then transferred into the testing solution. The electrode potential of the electrode was monitored, and the potential at 120 s was recorded. After each test, the electrode was removed from the testing solution, rinsed with 3.5 wt.% NaCl solution, and transferred into other testing solution with different TRCs. After all experiments, the electrode was rinsed with 3.5 wt.% NaCl solution and then ultrapure water, dried using a N_2_ flux, and stored in air at room temperature for the next measurements.

### 2.3. Potentiometric Measurements

A two-electrode cell, with the Fc-CPE serving as the working electrode and a saturated Ag/AgCl electrode with a saturated KCl inner solution serving as the reference electrode, was used for the potentiometric measurements. All the potentials reported in this work are referring to this Ag/AgCl reference electrode. After immersion of the Fc-CPE in solutions with different TRC concentrations or with different possible interference ions of different concentrations for 120 s, the open circuit potential (OCP) of the electrode was recorded on a CHI 604C electrochemical workstation (CHI instrument, Shanghai, China). All measurements were performed at 25 ± 0.2 °C.

## 3. Results

### 3.1. The Effect of Fc

The response time and stability of the electrode depends on the exchange current density of the electrochemical reaction occurring on the electrode/solution interface. For TRC/Cl^−^ reaction, its exchange current density is quite low. Hence, it is hard for the TRC/Cl^−^ reaction to attain electrochemical equilibrium and stable electrode potential. On the other hand, the high oxidation of HClO and ClO^−^ can oxidize electrode surface, resulting in passivation of the electrode surface in some cases. Hence, to prepare a direct potentiometric sensor based on TRC/Cl^−^ reaction is not easy. The exchange current density of Fc^+^/Fc electrochemical couple is one order of magnitude larger than that of TRC/Cl^−^. The higher exchange current density (*i*_ex_) of Fc^+^/Fc can shorten the time for the electrode to attain electrochemical equilibrium and stabile electrode potential. Moreover, Fc^+^/Fc does not oxidize the electrode surface, which is better for the electrode to remain stable and reproducible. There is an equilibrium between Fc^+^/Fc and TRC/Cl^−^ as

(4) $${2\mathrm{Fc}\;+\;\mathrm{Cl}}_{2}⇌{2\mathrm{Fc}}^{+}{+\;2\mathrm{Cl}}^{-}$$

The equilibrium constant of this equilibrium can be expressed as follows:

(5) $$K=\frac{c_{{\mathrm{Fc}}^{+}}^{2}c_{{\mathrm{Cl}}^{-}}^{2}}{c_{\mathrm{Fc}}^{2}\mathrm{TRC}}$$

(6) $$\frac{c_{{\mathrm{Fc}}^{+}}^{}}{c_{\mathrm{Fc}}^{}}=\frac{\sqrt{K\mathrm{TRC}}}{c_{{\mathrm{Cl}}^{-}}^{}}$$

According to the Nernst equation, the electrode potential can be written as follows:

(7) $$\varphi =\varphi^{⊖}+\frac{RT}{nF}\ln\frac{c_{{\mathrm{Fc}}^{+}}^{}}{c_{\mathrm{Fc}}^{}}$$

(8) $$\begin{array}{ll} \varphi & =\varphi^{⊖}+\frac{RT}{nF}\ln\frac{\sqrt{K\mathrm{TRC}}}{c_{{\mathrm{Cl}}^{-}}^{}}=\varphi^{⊖}+\frac{RT}{nF}\ln\frac{\sqrt{K}}{c_{{\mathrm{Cl}}^{-}}^{}}+\frac{RT}{nF}\ln{\mathrm{TRC}}^{1/2}\\ & =\varphi^{⊖}{}^{\prime }+\frac{RT}{2nF}\ln\mathrm{TRC} \end{array}$$

Because the equilibrium constant *K* of Equation (4) and the concentration of Cl^−^ in BW can be taken as constant, the second term in Equation (8) is a constant and can be merged into *φ*^⊖^′. According to Equation (8), the electrode potential increases with the increase of *c*_TRC_. Therefore, open circuit potential of the Fc-modified CPE is in linear proportion to lg*c*_TRC_, obeying the Nernstian equation.

### 3.2. The Effect of Composition

**Figure 2:**
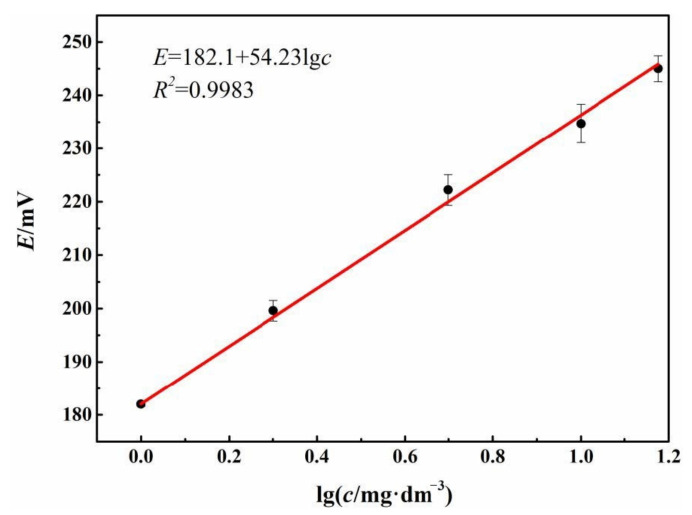
The linear relationship between open circuit potential (OCP) (*E*/mV) and logarithm of total residual chlorine (TRC) (lg (*c*/mg∙dm^−3^)) of No. 4 Fc-carbon paste electrode (CPE) electrode in 3.5 wt.% NaCl solution.

The effect of Fc on the potentiometric response of the electrode was investigated by changing the ratio of paraffin oil, graphite powder, and Fc, as listed in Table 1. The variation of OCP of the Fc-CPE electrode with different Fc content with logarithm of TRC (lg*c*) was measured and is shown in Table 1. The linearity of the relationships between OCP and lg*c* are listed in Figure 2.

According to Table 1, the potentiometric response of the electrode without Fc is not good. The potential of the electrode cannot become stable in reasonable time. After addition of Fc, a stable electrode potential can be achieved in less than 50 s. The OCP of the electrode is in linear proportion to logarithm of TRC within the TRC concentration range of 1–15 mg∙dm^−3^, showing a Nernstian relation as discussed in Section 3.1. According to Figure 2, the linear relationship of the No. 4 electrode is the best, with the slope of the linear part of 52.90 mV/decade which is close to the typical Nernstian slope of 59.16 mV/decade. Hence, the No.4 electrode with the masses of graphite power, paraffin oil, and Fc of 0.60 g, 0.25 g, and 0.15 g, respectively, was chosen for further study.

### 3.3. The Response Time and Reversibility of the Fc-CPE

The response speed of the electrode in 3.5 wt.% NaCl solution containing different TRC concentrations was studied.

**Figure 3:**
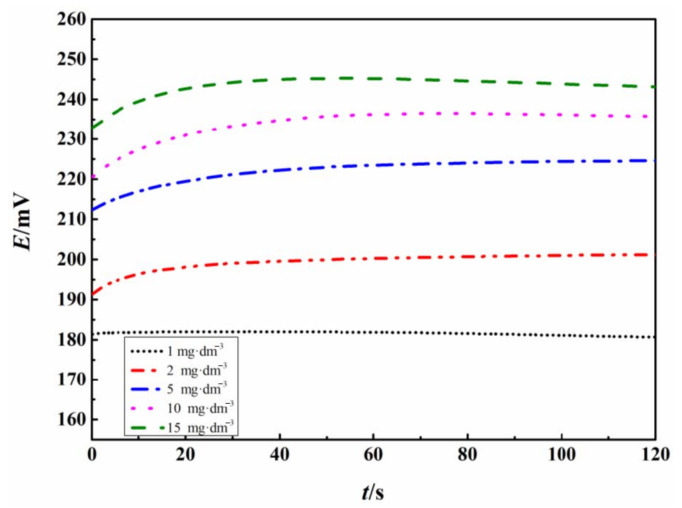
The variation of OCP (*E*) of the Fc-CPE electrode with immersion time in 3.5 wt.% NaCl solution containing different TRC concentrations.

According to Figure 3, after immersion of 40 s, the OCP of the electrode becomes stable. This respond speed is suitable for measurement of TRC.

**Figure 4:**
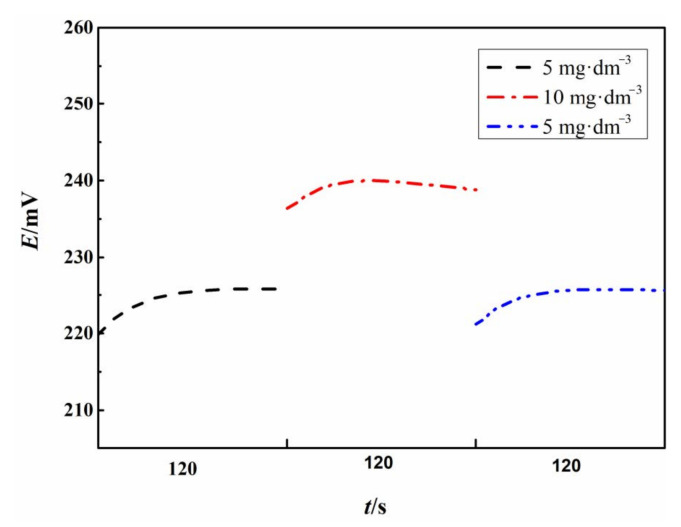
The potential recovery of the Fc-CPE after transfer between 3.5 wt.% NaCl solutions containing 5 mg∙dm^−3^ and 10 mg∙dm^−3^ TRC.

The reversibility of the Fc-CPE was studied by transferring the electrode in 3.5 wt.% NaCl solution containing 5 mg∙dm^−3^ TRC to the 3.5 wt.% NaCl solution containing 10 mg∙dm^−^^3^ TRC. The corresponding OCPs were recorded during the transfer and are shown in Figure 4.

According to Figure 4, the electrode potential of Fc-CPE can quickly restore to the initial value after rapid transfer between 3.5 wt.% NaCl solution containing different TRCs, showing good reversibility. There is no significant memory effect for the Fc-CPE.

### 3.4. Selectivity Studies

In BW, there are numerous cations such as K^+^, Mg^2+^, and Ca^2+^, and anions such as Br^−^, SO_4_^2−^, and HCO_3_^−^, which may interfere with the measurement of TRC using Fc-CPE sensor. The variation of OCP of the Fc-CPE with the concentration of these possible interference ions are recorded. The highest concentration of the possible interference ions is set according to the possible concentration in seawater.

**Figure 5:**
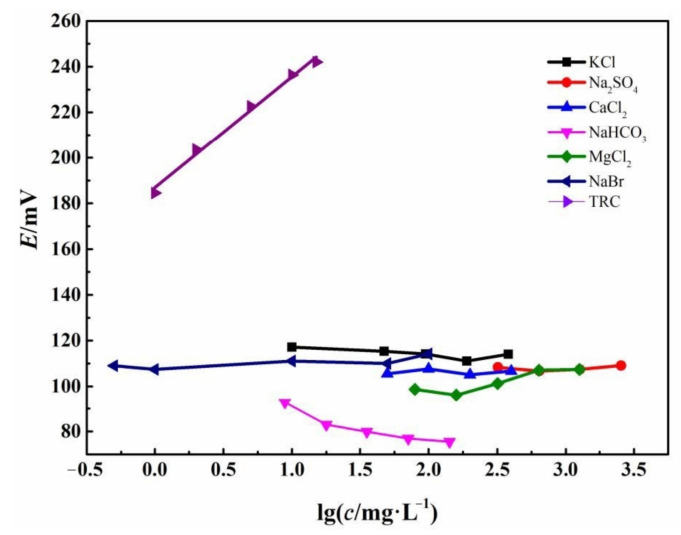
The OCP (*E*) of the Fc-CPE electrode in 3.5 wt.% NaCl containing possible interference ions with different concentrations.

According to Figure 5, the electrode potential of the Fc-CPE remains nearly unchanged with increasing of the interference ion concentration, with the only exception of NaHCO_3_. The other interference salts are all neutral. The OCP of Fc-CPE decreases with increasing of the NaHCO_3_ concentration, which can be attributed to Equations (9) and (10):

ClO^−^ + H_2_O + 2e^−^ → Cl^−^ + 2OH^−^ (9)

HClO + 2e^−^ → Cl^−^ + OH^−^ (10)

which suggests that the potential of Fc-CPE depends on pH of the solution. As the concentration of NaHCO_3_ increases, the pH of the solution increases, resulting in decreases in potential. However, once the seawater is pumped into the ballast water tank, the composition of BW does not change anymore. The possible interference can be excluded by setting a suitable potential baseline. Therefore, the selectivity of the Fc-CPE is good enough for monitoring TRC in the electrolytically-treated BW.

### 3.5. The Reproducibility, Lifespan, and Storage Stability of the Electrode

**Figure 6:**
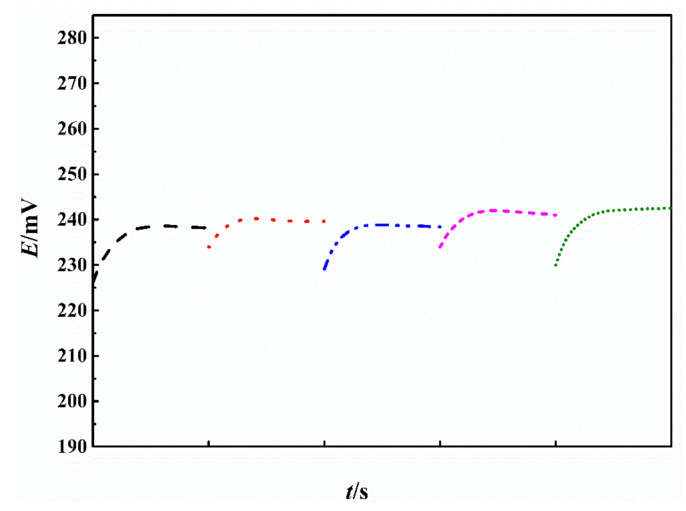
Potential variation of the five carbon paste electrodes of the same batch in 3.5 wt.% NaCl that contains 10 mg∙dm^−3^ TRC.

The reproducibility of the Fc-CPE electrodes was evaluated by measuring the electrode potential of five Fc-CPE electrodes of the same batch in 3.5 wt.% NaCl containing 10 mg∙dm^−3^ TRC. The electrode potentials of these five electrodes are shown in Figure 6.

According to Figure 6, the average electrode potential of these five electrodes is 238 mV, with the potential deviation less than ±1%, showing good reproducibility. The good reproducibility can be attributed to the easy fabrication of the carbon paste electrode and the good homogeneity of the past. The reproducibility of the Fc-CPE is better than that of other type of electrode reported elsewhere [29].

**Figure 7:**
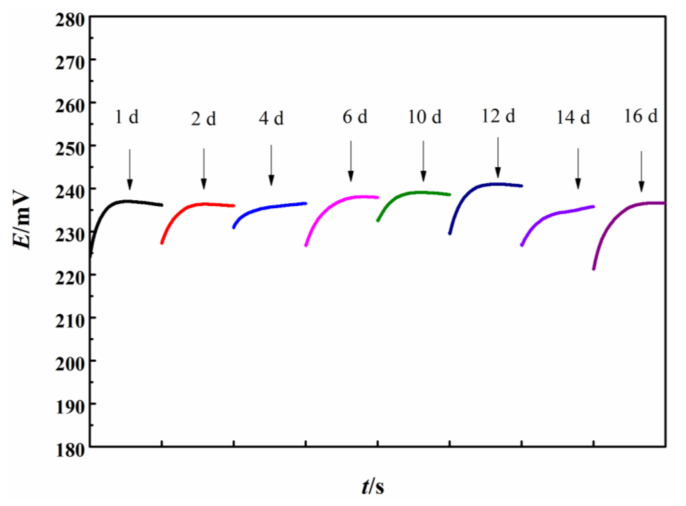
The OCP potential measured using the same Fc-CPE in 3.5 wt.% NaCl containing 10 mg∙dm^−3^ TRC, on different days.

For the sensors based on CPE, there may be some problems. For example, the volatility of the residual solvent used for preparation of CPE may affect the lifespan and stability of the sensor [38]. For Fc-modified CPE, the oxidation and dissolution of Fc during use may also result in uncertainties of the electrode. The lifespan and stability of the Fc–CPE electrode were evaluated by consecutive measurements. The OCP of a Fc-CPE in 3.5 wt.% NaCl solution containing 10 mg∙dm^−3^ TRC was recorded on different days, with the results shown in Figure 7.

In Figure 7, the same sensor was used for measurement every few days, and kept dry between measurements. After each test, the electrode was rinsed with 3.5 wt.% NaCl solution and then ultrapure water, and dried using a N_2_ flux. During the consecutive 16-day testing, the average potential of the Fc-CPE was 238 mV with the deviation of less than ±1.7%. This small deviation is acceptable for TRC measurement for the electrolytically-treated BW. This means that the Fc-CPE sensor can be used for TRC measurement with high reliability for more than two weeks.

The storage stability of the electrode was also studied by recording the OCP response of the Fc-CPE electrode to TRC after a storage at room temperature for 5 months.

**Figure 8:**
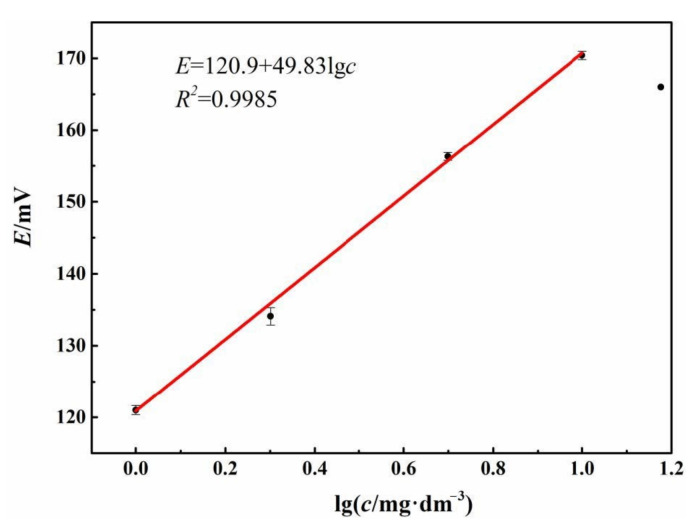
The linear relationship between OCP (*E*) and logarithm of TRC (lg*c*) of the Fc-CPE electrode in 3.5 wt.% NaCl solution after 5-month storage.

As shown in Figure 8, a good linear relationship between OCP and lg*c* with the intercept of 120.3 mV and slope of 50.27 mV/decade within the TRC concentration range of 1–10 mg∙dm^−3^ can be observed. The electrode potential measured in solution containing 15 mg∙dm^−3^ TRC deviates from the linear relation. Both the intercept and slope of the linear part become less than those of the newly-prepared electrode. These results suggest that some change has happened during the 5-month storage, but the change is not significant. Because the concerned concentration of TRC in BW is 6–10 mg∙dm^−3^, which lies within the TRC concentration range of 1–10 mg∙dm^−3^, the Fc-CPE after 5-month storage can be used after calibration.

## 4. Conclusions

A new potentiometric sensor based on ferrocene-modified carbon paste electrode was fabricated and used for testing TRC in simulated electrolytically-treated BW. Ferrocene is important for improved the performance of the sensor. The open circuit potential of the Fc-CPE is in linear proportion to the logarithm of TRC within the concentration range of 1 mg∙dm^−3^ to 15 mg∙dm^−3^. The reproducibility, reversibility, stability, and selectivity of the Fc-CPE sensor is quite good for TRC measurement. The lifespan and storage stability of the electrode are adequate. This sensor is suitable for the online monitoring of TRC for electrolytic treatment of BW.

## Figures and Tables

**Table 1:** The composition of the ferrocene (Fc)-modified carbon paste electrode.

No.	Composition (/g)			*R* ^2^	Respond Time (s)
	**Paraffin Oil**	**Graphite Powder**	**Fc**		
1	0.25	0.65	0	--	--
2	0.25	0.65	0.10	0.9583	45
3	0.3	0.6	0.10	0.9899	50
**4**	**0.25**	**0.60**	**0.15**	**0.9983**	**40**
5	0.2	0.6	0.2	0.9833	60
6	0.25	0.55	0.20	0.9819	42
7	0.20	0.55	0.25	0.9566	50

## Data Availability

Data sharing not applicable.
